# Our Animal Friends

**Published:** 1896-10

**Authors:** 


Our Animal Friends. A monthly magazine, published by The American Society for the Prevention of Cruelty to Animals. John P. Haines, President, 10 East Twenty-second Street, New York, N. Y. Subscription price, $1.00 per year. 10 cents a number.

The October number of this excellent journal contains several interesting articles. Among them we find "Facts and Fictions of Zoölogy," by the editor, a particularly valuable article; "The Bird Market of Paris," by Grace Gallatin; "Pets of Past and Present Times," by Esther Singleton; "Harmless Snakes," "A House of Bubbles" (illustrated), by Meredith Nugent; "The Little Lame Mouse," by Alice Howard Hilton, and others of less note. The prime object of this journal is to inculcate a love of and interest in animals and their habits, and so to suppress the disposition toward cruelty and ill-treatment of them.

